# HEATR1 Deficiency Promotes Chemoresistance via Upregulating ZNF185 and Downregulating SMAD4 in Pancreatic Cancer

**DOI:** 10.1155/2020/3181596

**Published:** 2020-05-26

**Authors:** Yuan Fang, Xu Han, Jianang Li, Tiantao Kuang, Wenhui Lou

**Affiliations:** Department of Pancreatic Surgery, Zhongshan Hospital, Fudan University, Shanghai 200032, China

## Abstract

**Objective:**

To discover the correlated gene with HEATR1 in regulating chemoresistance of gemcitabine.

**Methods:**

Gene chip analysis was performed to find out differential genes between HEATR1-KD and control groups. The top 20 genes were subjected to high-content screening, and functional assay was implemented. Gene expression profiling was carried out to find the downstream target. Immunohistochemistry and survival analysis were performed.

**Results:**

ZNF185 fold change (4.5285) was the most significant between the HEATR1-KD and control groups. Knocking down ZNF185 could promote the chemosensitivity, apoptosis, and proliferative inhibition, with SMAD4 significantly upregulated. Patients with high HEATR1 and SMAD4 or low ZNF185 exhibited better survival.

**Conclusion:**

HEATR1, ZNF185, and SMAD4 could affect the chemosensitivity of gemcitabine and may be the indicators of gemcitabine selection in the chemotherapy of pancreatic cancer.

## 1. Introduction

Pancreatic cancer is the most malignant GI tract cancers with the worst prognosis around the world. It is predicted to reach the second leading cause of cancer-related death by 2030 in the United States [1]. Gemcitabine has been the cornerstone chemotherapeutic agent of pancreatic cancer in the past 20 years [2]. Recently, the novel chemotherapeutic regimen including FOLFIRINOX (fluorouracil, leucovorin, irinotecan, and oxaliplatin) [3] or gemcitabine combined with albumin-bound paclitaxel [4] seemed to improve the survival but only limited to the patients with good performance because of the side effect of intolerable toxicity of FOLFIRINOX or paclitaxel. Therefore, gemcitabine is still the first-line chemotherapeutic agent for pancreatic cancer, and understanding the mechanisms of the resistance will provide significant clinical strategy. Unfortunately, the mechanism of gemcitabine resistance has not been fully elucidated although the previous research was focused on the molecular and cellular changes including gemcitabine metabolism enzymes, inhibition of the apoptotic pathway, activation of the cancer stem cells (CSC), or epithelial-to-mesenchymal transition (EMT) [5].

HEAT repeat-containing protein 1 (HEATR1) contains HEAT repeats, initially found in some proteins including huntingtin, elongation factor 3, and the PR65/A subunit of phosphatase 2A [6]. The human HEATR1 gene is located on chromosome 1q43 and encodes a high molecular weight (236 KDa) protein with 2144 amino acids. Our team discovered the effect of HEATR1 on the chemosensitivity of gemcitabine and published the original research on *Cancer Research* in 2016, explaining the possible mechanism in that HEATR1 enhanced the chemosensitivity to gemcitabine by facilitating the interactions between AKT and PP2A and promoting Thr308 dephosphorylation [7]. In this study, our team aim is to discover novel functional genes correlated with HEATR1 in sensitizing pancreatic cancer cells to gemcitabine, which may help to search for a new therapeutic target and improve the efficacy of gemcitabine in the pancreatic cancer.

## 2. Materials and Methods

### 2.1. Ethical Statement

The study protocol was approved by the Independent Ethics Committee at Zhongshan Hospital, Fudan University. Written informed consent forms were signed by all the participating patients, and all the experiments were in accordance with the Declaration of Helsinki revised in 2013 [8].

### 2.2. Cell Lines

Human pancreatic cancer cell lines PANC-1, SW 1990, MIA-PaCa2, Patu-8988, and Capan-1 were purchased from ATCC, and the identification of all the cell lines was confirmed by STR profiling at GeneChem Company (Shanghai, China). PANC-1 and MIA-PaCa2 were cultured in a medium containing high-glucose Dulbecco's modified Eagle medium (DMEM) (Gibco, Grand Island, New York, USA) supplemented with 10% fetal bovine serum (Gibco), 100 U/mL penicillin, and 100 U/mL streptomycin (Gibco) in a humidified 37°C and 5% CO_2_ incubator, while Patu-8988, Capan-1, and Sw 1990 cell lines were cultured with the RPMI-1640 medium (Gibco) instead of DMEM.

### 2.3. Quantitative Real-Time PCR

Total RNA was isolated using the Trizol reagent (Invitrogen, Carlsbad, CA, USA) from human pancreatic cancer cell lines. Standard cDNA synthesis reactions were implemented using the M-MLV Reverse Transcriptase kit (Promega, USA) following the instructions [9]. For qRT-PCR analysis, reverse transcribed products were amplified using SYBR Premix Ex Taq (TaKaRa, Japan). PCR reactions were carried out using the ABI 7500 Real-Time PCR (Applied Biosystems, CA, USA) and repeated three times. Relative mRNA levels were normalized to GAPDH, and the relative expression of transcripts was analyzed by using the 2^−△△Ct^ methods. The following primers were used: human HEATR1 (Gene accession no. NM_018072): 5-TTCACTTGTCGCCTTACTTCC-3 (forward) and 5-CCAGAACCATCTGTGCTTTGA-3 (reverse); human ZNF185 (Gene accession no. NM_007150):5-GATCCGAGACTGTCCAAAGAT-3 (forward) and 5-AATGGTGTCACGGTGAATGA-3 (reverse); human SMAD4 (Gene accession no. NM_005359):5-ACGAACGAGTTGTATCACCTGG-3 (forward) and 5-TGCACGATTACTTGGTGGATG-3 (reverse); human GAPDH: 5-TGACTTCAACAGCGACACCCA-3 (forward) and 5-CACCCTGTTGCTGTAGCCAAA-3 (reverse).

### 2.4. Western Blot Analysis

Total proteins extracted with the cell lysate buffer and protease inhibitors were separated using 10% sodium dodecyl sulfate-polyacrylamide gel electrophoresis (SDS-PAGE) and electrotransferred onto polyvinylidene difluoride membranes (Millipore, Billerica, MA, USA). Membranes were incubated with the primary antibody: HEATR1 (1 : 10000, ab241610, Abcam, CA, USA), FLAG (1 : 2000, F1804, Sigma, USA)， SMAD4 (1 : 1000, AF2097, R&D systems, MN, USA), and GAPDH (1 : 2000, sc-32233, Santa Cruz, Texas, USA) overnight after being blocked with 5% nonfat milk in TBS-T. Protein expression was detected with the Image System using Tanon 5200 (Tanon, Shanghai, China).

### 2.5. Stable Knockdown of HEATR1 and ZNF185

The GV115 puromycin lentiviral vectors were designed and constructed by GeneChem Co., Ltd. (Shanghai, China). The human HEATR1 siRNA target sequence was GCTGAACAAGTCCGAATAGAA. The GV115 puromycin lentiviral vectors were used as controls. Stably transfected clones for HEATR1 were validated by western immunoblot analysis. The human ZNF185 siRNA target sequence was TCCAAAGATTACCCTAGAA, and the target sequence was cloned into the GV115 lentiviral vector. ZNF185 knockdown efficiency was validated by qPCR in PANC-1 cells, and western blot validation was carried out in 293T cells expressing 3 × FLAG-ZNF185 fused protein. After knockdown efficiency validation, stable shHEATR1 cells were transfected with the shZNF185 lentivirus, and functional experiments were arranged.

### 2.6. Cell Proliferation Assay

For the cell proliferation assay, 5000 cells in every well dispensed in 100 *µ*l aliquots were seeded in a 96-well plate, and the viable cells were counted after 72 hours after treated with gemcitabine (10 *µ*M). Cells were incubated in 10% CCK-8 (Dojindo Molecular Technologies, Gaithersburg, MD, USA) diluted in a normal culture medium for an additional 2 h. For estimating the viable cells and IC50 values, the absorbance at a wavelength of 450 nm was measured in every well. Finally, the cells were fixed with 1% paraformaldehyde for 30 minutes and stained with 0.1% (w/v) crystal violet for 30 min [10]. The numbers of cell colonies were counted using the Image-Pro Plus 5.0 software (Media Cybernetics, Bethesda, MD, USA).

### 2.7. Cell Apoptosis Assay

Apoptosis was measured by using the Annexin V-APC Kit (BD Bioscience) after 72 hours after treated with gemcitabine (10 *µ*M). Briefly speaking, the cells were harvested with trypsin, washed twice with ice-cold PBS, and resuspended 1 × binding buffer. Then, 10 ul of annexin V-APC was added into 200 ul of cell suspensions. After incubation for 15 mins, the population study of the target cohort was performed by a FACS Aria II flow cytometer (BD Bioscience) [11].

### 2.8. Gene Chip Analysis

Total RNAs were extracted from cells and then subjected to gene chip analysis to find out differential expression genes (DEGs) (fold change >1.5 and *P* value < 0.05) between the two groups (HEATR1-KD vs NC). DEGs were further analyzed, and 30 genes were selected. Selection principles were as follows: (1) literature review was carried out to ensure that the selected DEGs were not reported with functions previously in pancreatic cancer; (2) transmembrane protein-encoding genes were ruled out due to their low knockdown efficiency; (3) fold change of selected DEGs was larger than 2.4. These 30 selected genes were tested for their expression levels in HEATR1-KD PANC-1 cells using quantitative PCR.

### 2.9. High-Content Screening

The high-content screening (HCS) was carried out by the method mentioned in the previous reports [12]. In brief, PANC-1 cells were transfected with HEATR1 knockdown lentivirus (HEATR1-KD cells). Then, different RNAi sequences targeting 20 genes were transfected to HEATR1-KD cells using the lentiviral vector containing GFP tags to monitor cell viability. The GFP-positive cells were cultured and observed for 5 days. Live cells were counted and analyzed for 5 days using the HCS instrument software (CQ1, Yokogawa). Differentially expressed genes from HCS screening were uploaded to Ingenuity Pathway Analysis (IPA, Ingenuity System) for pathway analysis and molecular pathway identification.

### 2.10. Immunohistochemical Analysis

A tissue microarray was constructed following the standard tissue protocols as described previously [13]. Primary antibodies were HEATR1 (rabbit monoclonal; 1 : 500; Abcam, USA), ZNF185 (rabbit polyclonal; 1 : 1000: Invitrogen, USA), and SMAD4 (rabbit monoclonal; 1 : 100; Abcam, USA) followed by incubation with the secondary antibody. The positive staining was measured by a computerized image system including a Leica-CCD camera connected to a Leica-DM-IRE2 microscope. Pictures of representative fields were captured by the Leica QWin Plus v3 software. The specimens with negative or weak intensity (±) of HEART1, ZNF185, and SMAD4 were graded as low expression, while those with moderate or strong (++/+++) were graded as high expression.

### 2.11. Patients' Specimens and Follow-Up

From January 12, 2012, to March 3, 2017, the same pancreatic surgical group in our hospital performed radical resection for pancreatic cancer on 80 consecutive patients. Overall survival (OS) was defined as the interval between the date of surgery and death or the last follow-up day. The last follow-up day was 1 November 2019. None of the patients received any preoperative treatment, and all of the patients received a postoperative chemotherapeutic regimen of six cycles of standard gemcitabine [14]. Clinicopathologic features were staged according to the 2002 International Union Against Cancer's tumor-node-metastasis (TNM) classification system [15].

### 2.12. Statistical Analysis

SPSS 20.0 (Chicago, IL, USA) was used for statistical analysis. Qualitative variables were compared using the Pearson *χ*^2^ test or the Fisher exact test. Quantitative data were recorded as mean ± SD and compared using the *t*-test or one-way ANOVA. Significance was determined at *P* < 0.05. Kaplan–Meier analysis and log-rank test were used to compare the survival. The Cox regression model was used to perform multivariate survival analysis.

## 3. Results

### 3.1. HEATR1 Promotes the Chemosensitivity to Gemcitabine in Pancreatic Cell Lines

**Figure 1:**
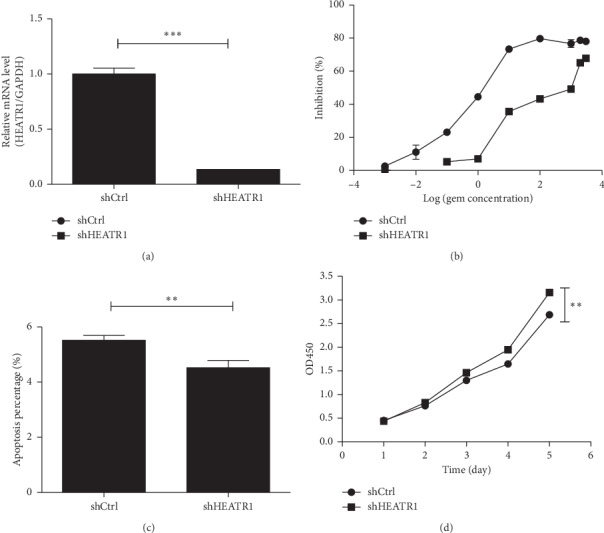
HEATR1 promotes the chemosensitivity to gemcitabine in pancreatic cell lines. (a) HEATR1 knockdown in PANC-1. (b) Increased chemoresistance of PANC-1 to gemcitabine after HEATR1 knockdown. (c) Apoptotic percentage decreased after HEATR1 knockdown when treated with gemcitabine. (d) Proliferation increased on day 5 dramatically after HEATR1 knockdown when treated with gemcitabine.

After we examined the HEATR1 mRNA levels in 5 pancreatic cell lines, we firstly knocked down HEATR1 expression in the pancreatic cancer cell line PANC-1 using the RNA interference technique through the lentivirus vector (shHEATR1) and the scrambled sequence as the negative control (shCtrl) (Figure 1(a)). We found that the chemoresistance pancreatic cancer to gemcitabine was much more significant after HEATR1 knockdown (Figure 1(b)), and the apoptotic percentage decreased (Figure 1(c)) when proliferation increased dramatically (Figure 1(d)) on day 5 after HEATR1 was knockdown.

### 3.2. Gene Chip Analysis of Differentially Expressed Genes and HCS Screening after HEATR1 Knockdown

**Figure 2:**
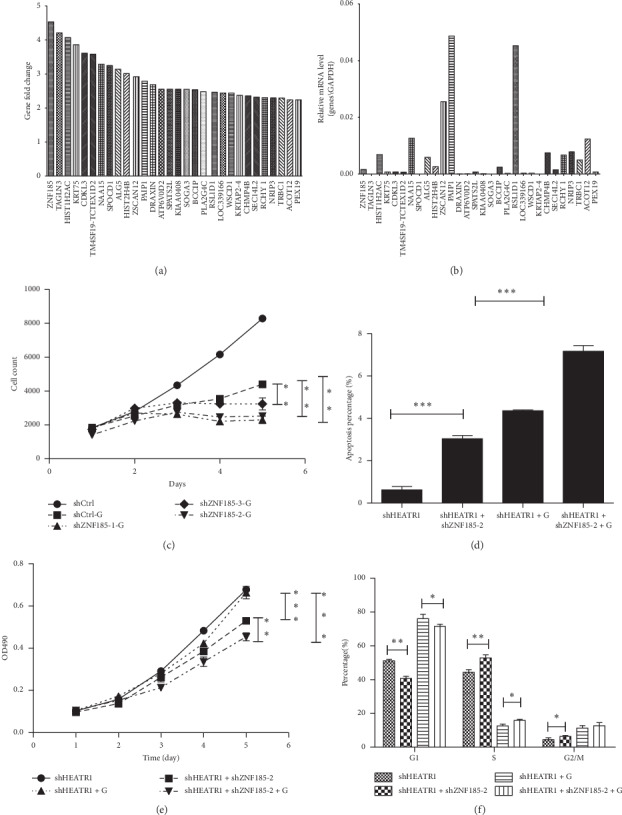
Gene chip analysis of differentially expressed genes and HCS screening after HEATR1 knockdown. (a) Differential expression fold change (4.5285) of ZNF185 was the most significant in the selected 30 genes between the HEATR1-KD and control groups in gene chips. (b) Quantitative PCR of the selected 30 genes. (c) In HCS screening, knocking down ZNF185 in the pancreatic cancer cells could promote the chemosensitivity of gemcitabine; confirmation of chemosensitive function of ZNF185 by observing the increased apoptotic percentage (d) and proliferative inhibition (e) when HEATR1 and ZNF185 were both knocked down. (f) After knocking down ZNF185, the pancreatic cancer cells in the S phase and the G2/M phase increased, while the cells in the G1 phase decreased.

The top 20 out of 30 selected genes with high expression levels were subjected to high-content screening (HCS) in HEATR1-KD cells to testify whether they could affect cell proliferation activity when knocking down their expressions, with or without gemcitabine treatment, respectively. After the selection from the gene chips and the bioinformatical analysis, we discovered ZNF185, whose fold change (4.5285) was the most significant in these 20 genes between the HEATR1 knocking down and control groups (Figure 2(a)). Then, we tested the expression levels of these 30 selected genes in HEATR1-KD PANC-1 cells using quantitative PCR (Figure 2(b)). By high-content screening (HCS), we confirmed that knocking down ZNF185 in the pancreatic cancer cells could promote the chemosensitivity of gemcitabine (Figure 2(c)).

### 3.3. ZNF185 Enhances the Chemoresistance to Gemcitabine in HEATR1 Knockdown Pancreatic Cancer Cells

We confirmed the chemosensitive function of ZNF185 by observing the elevation of apoptotic percentage (Figure 2(d)) and proliferative inhibition (Figure 2(e)) of pancreatic cancer cells after knocking down both HEATR1 and ZNF185 and treated with gemcitabine. Moreover, we found that after knocking down ZNF185, the pancreatic cancer cells in the *S* phase and the G2/M phase increased, while the cells in the G1 phase decreased (Figure 2(f)), both of which indicated that the cellular proliferation was inhibited in the *S* phase.

### 3.4. Expression of SMAD4 Increases in the ZNF185 Knockdown Pancreatic Cancer Cells

**Figure 3:**
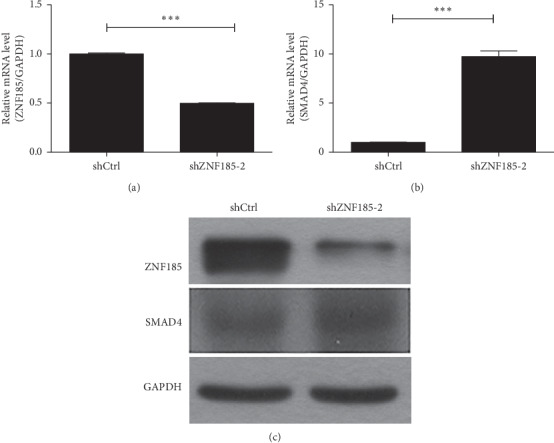
Expression of SMAD4 increased in the ZNF185 knockdown pancreatic cancer cells. (a) qRT-PCR of ZNF185 and (b) SMAD4 when ZNF185 was knocked down. (c) Western blots of ZNF185 and SMAD4 when ZNF185 was knocked down.

To determine the possible downstream genetic target of ZNF185, we performed gene expression profiling of PANC-1 cells transduced with either control or shZNF185 lentivirus. Genes were upregulated and downregulated; red denotes the upregulated genes, and green denotes the downregulated genes (Supplementary Materials (available here)). Enriched canonical pathways were analyzed using IPA (Ingenuity Pathway Analysis). We discovered that the SMAD4 significantly increased. The expression of qRT-PCR (Figures 3(a) and 3(b)) and western blots (Figure 3(c)) for ZNF185 and SMAD4 were consistent with the gene expression profiling data.

### 3.5. Expression of HEATR1, ZNF185, and SMAD4 in the Pancreatic Cancer Tissues and Correlation with the Survival

**Figure 4:**
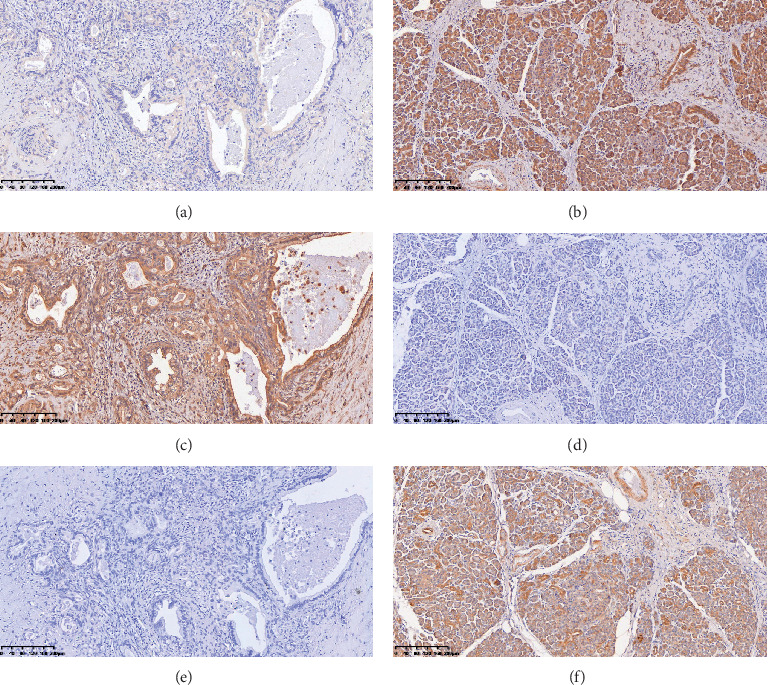
Expression status in pancreatic ductal adenocarcinoma. Representative images of expression of HEATR1 in cancer (a) and normal (b) tissues. Representative images of expression of ZNF185 in cancer (c) and normal (d) tissues. Representative images of expression of SMAD4 in cancer (e) and normal (f) tissues.

We analyzed the expression levels in pairs of pancreatic cancer and normal pancreatic tissues. Representative IHC of HEATR1, ZNF185, and SMAD4 is shown in Figure 4. HEATR1 showed mainly low expression of 80% (64/80) in cancer and high expression of 65% (52/80) in normal pancreas (Figures 4(a) and 4(b)). We also observed that ZNF185 was mainly low (56.3%, 45/80) in the normal pancreas and high (51.2%, 41/80) in cancer (Figures 4(c) and 4(d)). SMAD4 was low (50%, 40/80) in the normal pancreas and mainly low (73.8%, 59/80) in cancer (Figures 4(e) and 4(f)). The *χ*^2^ test showed significant differences in the expression of HEATR1 (*χ*^2^ = 37.143, *P* < 0.001), ZNF185 (*χ*^2^ = 20.077, *P* < 0.001), and SMAD4 (*χ*^2^ = 23.309, *P* < 0.001) between the cancer and the normal pancreas.

When analyzing the relationships between HEATR1, ZNF185, and SMAD4 expression grading and the clinicopathological factors (Table 1), we found that a higher HEATR1 expression grading showed a significant correlation with normal CA19-9 (*χ*^2^ = 9.879, *P*=0.002) and higher differentiation (*χ*^2^ = 9.135, *P*=0.002), while higher ZNF185 expression was correlated with higher CA19-9 (*χ*^2^ = 4.121, *P*=0.042) and poorer differentiation (*χ*^2^ = 31.223, *P*=0.001), and higher SMAD4 was correlated with normal CA19-9 (*χ*^2^ = 7.944, *P*=0.005) and higher differentiation (*χ*^2^ = 9.399, *P*=0.002).

**Figure 5:**
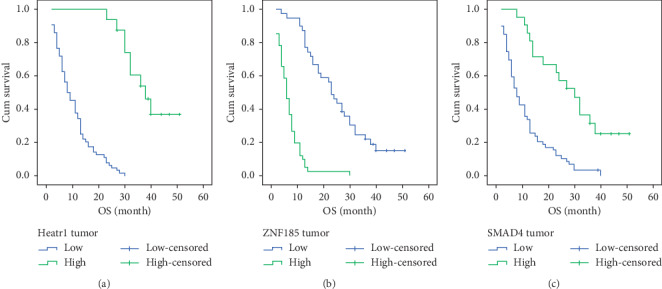
Expression of HEATR1, ZNF185, and SMAD4 in the pancreatic cancer tissues and correlation with the survival. (a) Univariate analysis of HEATR1 expression and OS. (b) Univariate analysis of ZNF185 expression and OS. (c) Univariate analysis of SMAD4 expression and OS.

The median survival time of the cohort was 12 months. After the patients were treated with gemcitabine chemotherapy, patients with higher HEATR1 (*P* < 0.001) and SMAD4 (*P* < 0.001) staining or lower ZNF185 (*P* < 0.001) staining exhibited better overall survival (Figure 5). From the multivariate survival analysis, we observed that high TNM staging, poorer differentiation, higher staining of HEATR1, or lower staining of ZNF185 in pancreatic cancer were the independent prognostic factors of pancreatic cancer patients (Table 2).

## 4. Discussion

Gemcitabine has long been the first-line chemotherapeutic agent for pancreatic cancer during the past 20 years, but the clinical response was always unsatisfactory mainly caused by the chemoresistance within the several weeks after starting the treatment [16]. The possible mechanism of the chemoresistance in the previous study included inadequate transmembrane transport of the agent due to hypovascularity [17], antiapoptotic pathways [18], and epithelial-mesenchymal transition [19]. In our previous study, we discovered that downregulation of HEATR1 in pancreatic cancer causes resistance to gemcitabine. We hypothesized that HEATR1 promotes gemcitabine efficacy through inhibiting AKT phosphorylation [7]. But, whether is there any functional downstream gene of HEATR1 that synergistically regulates the chemosensitivity of gemcitabine is still an interesting further objective.

Here, we found that knocking down ZNF185 could synergistically promote the chemosensitivity of gemcitabine in pancreatic cancer cells. ZNF185 belonging to the ZNF family, located on the DXS52 region of the long arm of chromosome Xq28, is one kind of the actin-cytoskeleton-associated Lin-l 1, Isl-1, and Mec-3 (LIM) domain-containing protein [20]. The knowledge about the function of ZNF185 is still lacking, while some previous studies have shown its role in cell proliferation, cell differentiation, and cell apoptosis in the prostate, lung, and head and neck squamous cell carcinoma [21]. Our observation is the first to identify a relationship between ZNF185 regulation and gemcitabine chemosensitivity of pancreatic cancer. Moreover, we also preliminarily discovered that knocking down ZNF185 served as increasing the chemosensitivity via SMAD4 in pancreatic cancer which was confirmed by *in vitro* and *in vivo* analysis. The tumor suppressor gene SMAD4 is first referred to as pancreatic cancer deletion gene4 (DPC4) because the deficiency in its expression was first discovered in pancreatic cancer [22]. A previous study showed that genetic alterations or homozygous deletion of SMAD4 can influence the normal signalling of the TGF-*β* pathway and uncontrolled cell growth and pancreatic tumorigenesis [23], and low expression of SMAD4 is correlated with poor survival in pancreatic cancer, but there are very few studies on SMAD4 with chemosensitivity of gemcitabine in pancreatic cancer [24]. Besides, the relevance of our study was confirmed in the samples of pancreatic cancer patients, and we demonstrated that HEATR1, ZNF185, and SMAD4 expression are significantly associated with the prognosis with pancreatic cancer in the patients treated with gemcitabine chemotherapy. Therefore, it is possible that patients with high IHC staining of HEATR1 or low staining of ZNF185, which indicate the chemosensitive markers, are suitable for gemcitabine treatment and may have better prognosis.

Our study also had some limitations. First, all of the functional assays were implemented in the HEATR1-KD cells, while we failed to perform the overexpression of HEATR1 because of the large molecular weight of HEATR1. Second, in the past 5 years, there were increasing number of the different chemotherapeutic regimens for pancreatic cancer, including gemcitabine monotherapy or combination with other agents (e.g., oxaliplatin and albumin-bound paclitaxel), or even more toxic regimen of FOLFIRINOX. Although these regimens showed encouraging prognostic benefit in the USA or European patients [25], most of the Chinese patients still cannot endure the toxicity of FOLFIRINOX or Abraxane because of the side effects such as diarrhea [26]. Besides, according to the NCCN guidelines in 2019, no definite standard has been established in the adjuvant treatment of pancreatic cancer so far. Chemotherapy alone with gemcitabine (category 1), 5-FU/leucovorin (category 1), gemcitabine/capecitabine (category 1), or continuous infusion 5-FU is listed in the guidelines as options for adjuvant treatment. Therefore, gemcitabine is still the cornerstone chemotherapy agent of pancreatic cancer in the East Asian, especially the Chinese pancreatic patients. Whether the HEATR1-ZNF185-SMAD4 pathway exerts the same chemosensitive mechanism in these agents will need further research.

Taken together, it is the first time to reveal that HEATR1, ZNF185, and SMAD4 are correlated in the chemosensitivity of gemcitabine in pancreatic cancer. These results require further studies with a larger patient cohort to confirm and chemoresistance mechanism for pancreatic cancer. A better understanding of the causes of gemcitabine chemoresistance is critical to the development of novel comprehensive treatment strategies for pancreatic cancer.

## Figures and Tables

**Table 1:** Relationship between HEATR1, ZNF185, and SMAD4 expression and clinicopathological features of pancreatic cancer.

Group	HEATR1 expression		*P* value	ZNF185 expression		*P* value	SMAD4 expression		*P* value
	Low	High		Low	High		Low	High	
Gender									
Female	28	11	0.074	19	22	0.659	34	7	0.056
Male	36	5		20	19		25	14	
Age (year)									
<60	24	8	0.361	15	17	0.784	23	9	0.756
≥60	40	8		24	24		36	12	
CA19-9 (U/ml)									
<37	9	8	**0.002**	12	5	**0.042**	8	9	**0.005**
≥37	55	8		27	36		51	12	
Tumor diameter (cm)									
<3	17	4	0.899	10	11	0.904	17	4	0.382
≥3	47	12		29	30		42	17	
TNM staging									
IA∼IIA	35	13	0.052	26	22	0.235	34	14	0.468
IIB∼IV	29	3		13	19		25	7	
Lymphatic positive									
No	43	13	0.272	28	28	0.733	42	14	0.698
Yes	21	3		11	13		17	7	
Tumor location									
Head	45	7	0.046	23	29	0.270	42	10	0.052
Body and tail	19	9		16	12		17	11	
Differentiation									
High and medium	25	13	**0.003**	31	7	**0.001**	22	16	**0.002**
Poor	39	3		8	34		37	5	
Vessel invasion									
No	54	14	0.754	35	33	0.247	49	19	0.413
Yes	10	2		4	8		10	2	
Vessel metastasis									
No	40	13	0.156	26	27	0.939	36	17	0.097
Yes	24	3		13	14		23	4	
Perineural invasion									
No	45	12	0.711	26	31	0.377	43	14	0.589
Yes	19	4		13	10		16	7	

The bold values indicate *P* values less than 0.05.

**Table 2:** The clinicopathological stigma and multivariate survival analysis.

Variables	Patients (*n* = 80)	OS		
		Univariate *P* value	Multivariate *P* value	Hazard ratio (95% CI)
Gender				
Female	39	0.532		
Male	41			
Age (years)				
<60	32	0.577		
≥60	48			
CA19-9 (U/ml)				
<37	17	**0.005**	0.202	
≥37	63			
Tumor diameter (cm)				
<3	21	0.847		
≥3	59			
TNM staging				
IA∼IIA	48	**<0.001**	**0.027**	2.597 (1.113–6.057)
IIB∼IV	32			
Lymphatic positive				
No	56	**0.014**	0.439	
Yes	24			
Tumor location				
Head	52	0.301		
Body and tail	28			
Differentiation				
High and medium	38	**<0.001**	**<0.001**	4.257 (2.105–8.609)
Poor	42			
Vessel invasion				
No	68	0.158		
Yes	12			
Vessel metastasis				
No	53	0.266		
Yes	27			
Perineural invasion				
No	57	0.523		
Yes	23			
HEATR1 tumor expression				
Low	64	**<0.001**	**<0.001**	0.099 (0.027–0.360)
High	16			
ZNF185 tumor				
Expression	39	**<0.001**	**0.004**	2.765 (1.385–5.519)
Low	41			
High				
SMAD4 tumor				
Expression	59			
Low	21	**<0.001**	0.355	
High				

The bold values indicate *P* values less than 0.05.

## Data Availability

The data used to support the findings of this study are available from the corresponding author upon request.

## Supplementary Materials

Supplementary Materials Gene expression profiling of PANC-1 cells transduced with either control or shZNF-185 lentivirus. Genes were upregulated, and genes were downregulated; red denotes the upregulated genes, and green denotes the downregulated genes. H3608-1, H3608-2, and H3608-3 indicate 3 repeated gene profiling after transduced with shCtrl lentivirus. H3609-1, H3609-2, and H3609-3 indicate 3 repeated gene profiling after transduced with ShZNF185.
