# Supplementary figures and images for: HEATR1 Deficiency Promotes Chemoresistance via Upregulating ZNF185 and Downregulating SMAD4 in Pancreatic Cancer
Source: J Oncol. 2020 May 26;2020:3181596. doi: 10.1155/2020/3181596 (PMC7271247; doi:10.1155/2020/3181596)

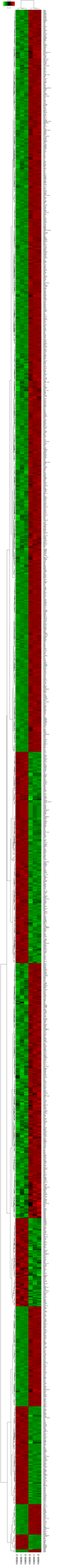

Supplement: Supplementary Materials — Gene expression profiling of PANC-1 cells transduced with either control or shZNF-185 lentivirus. Genes were upregulated, and genes were downregulated; red denotes the upregulated genes, and green denotes the downregulated genes. H3608-1, H3608-2, and H3608-3 indicate 3 repeated gene profiling after transduced with shCtrl lentivirus. H3609-1, H3609-2, and H3609-3 indicate 3 repeated gene profiling after transduced with ShZNF185. [file 3181596.f1.pdf]
